# A Novel 1259 bp Intragenic Deletion in the *GJB2* Gene in a Mexican Family with Congenital Profound Hearing Loss

**DOI:** 10.3390/audiolres15050111

**Published:** 2025-09-02

**Authors:** David Oaxaca-Castillo, Laura Taño-Portuondo, Montserrat Rodríguez-Ballesteros, Gerardo Pérez-Mendoza, Igrid García-González, Jorge Canto-Herrera, María Domínguez-Ruiz, Doris Pinto-Escalante, Orlando Vargas-Sierra, Damaris Estrella-Castillo, Paola López-González, Javier E. Sosa-Escalante, Ignacio del Castillo, Lizbeth González-Herrera

**Affiliations:** 1 Laboratorio de Genética, Centro de Investigaciones Regionales, Unidad Biomédica, Universidad Autónoma de Yucatán, Calle 43 #613 x 96, Colonia Inalámbrica, Mérida 97225, Mexico; 2 Servicio de Genética, Hospital Universitario Ramón y Cajal, IRYCIS, 28034 Madrid, Spain; 3 Centro de Investigación Biomédica en Red de Enfermedades Raras (CIBERER), 28034 Madrid, Spain; 4 Unidad Universitaria de Rehabilitación, Facultad de Medicina, Universidad Autónoma de Yucatán, Avenida Itzáes No. 498, Centro, Mérida 97000, Mexico; 5 Diagnósticos Moleculares y Genéticos, DIMYGEN Laboratorio S.C.P., Calle 78 No. 578 x 13-1 y 128, Residencial Pensiones, Mérida 97217, Mexico

**Keywords:** *GJB2* gene, connexin-26, hearing loss, intragenic deletion, Sanger sequencing

## Abstract

Hearing loss is a genetically heterogeneous sensory defect for which biallelic pathogenic variants in the *GJB2* gene are a frequent cause. Here, we report a novel intragenic large deletion in *GJB2* in a Mayan family with several members affected by congenital non-syndromic hearing loss. The analysis of the *GJB2* gene in the proband was performed through Sanger sequencing. A novel homozygous 1259 bp deletion in *GJB2* was identified, starting at nucleotide 248 of the coding region and ending at nucleotide 825 of the 3′-UTR (g.20188077_20189335del). Bioinformatic tools were used to predict the structural impact of the variant. This deletion would result in a truncated protein of 86 amino acids, p.(Phe83Cysfs*5), disrupting several critical domains of the connexin-26 protein. We developed an endpoint-PCR assay to test for the deletion. It was present homozygously in all affected siblings and was absent in 153 ethnically matched controls with normal hearing. Both parents and two unaffected siblings were heterozygous carriers, consistent with an autosomal recessive inheritance pattern. The identification of this novel large deletion expands the spectrum of *GJB2* pathogenic variants causing non-syndromic hearing loss, and it is of concern to *GJB2* screening methods that rely primarily on Sanger sequencing for its coding region.

## 1. Introduction

Hearing impairments are a heterogeneous group of disorders with a significant impact on quality of life due to the disability that they generate. According to the World Health Organization (WHO), 5% of the world’s population suffers from some degree of disabling hearing loss [1]. In Mexico, deafness is the fourth leading cause of disability [2], with an estimated 3 in every 1000 live births being affected [3]. Particularly in Yucatán, the average rate of deafness is 4.4 per 1000 inhabitants [4].

Genetic factors are responsible for more than 50% of neonatal hearing loss, with 15% being syndromic and 35% non-syndromic [5,6]. Diagnosing non-syndromic hearing loss can be complicated due to the high heterogeneity of loci and genetic variants that can be found, as well as factors such as inbreeding often observed among affected individuals. Pathogenic variants in the *GJB2* gene, which encodes the intercellular gap junction protein connexin 26 (Cx26), are the main genetic factor associated with non-syndromic hearing loss [7], with spectra of variants varying among different ethnic groups [8]. Although more than 277 pathogenic and likely pathogenic variants in the *GJB2* gene have been described [9], only nine large deletions at the DFNB1 locus have been shown to contribute to hearing loss. They either remove the whole *GJB2* gene or delete an upstream region containing a cis-acting regulatory element that is essential for the expression of *GJB2*. Some of these deletions also remove the *GJB6* gene (connexin 30) [10].

Although genetic-molecular studies conducted in Mexico to determine the etiology of congenital hearing loss are scarce, they have detected the presence of homozygous or compound heterozygous pathogenic variants in the *GJB2*, *GJB6*, *SLC26A4*, and *CDH23* genes [11,12,13]. In particular, variants in the *GJB2* gene are the most prevalent (9.6–16.4%) [5], with 30 variants reported, 26 of which were classified as pathogenic [14,15,16,17,18,19].

Chicán (Chi’Kaan in the Mayan language) is a small rural community of Mayan origin with approximately 705 inhabitants located in the municipality of Tixmehuac in Yucatán, southeastern México [20]. This community is relatively isolated with limited access to health services and a high rate of consanguinity [21]. A significant number of deaf people have been documented in this community, accounting for approximately 2.36% of its inhabitants [22], in contrast with the national deafness rate in Mexico (0.44%). There are no data on pathogenic variants in the *GJB2* gene in the Mexican Mayan population; however, the pathogenic variant c.131G > A (p.Trp44*) has been reported as the most common variant in the Guatemalan population of Mayan descent, with an allele frequency of 0.079 [23].

Here, we report a novel large intragenic deletion within the *GJB2* gene in a Mayan family with several members affected by congenital non-syndromic hearing loss. This study expands the spectrum of *GJB2* pathogenic variants causing hearing loss, and it is of concern to *GJB2* screening methods that rely primarily on sequencing its coding region.

## 2. Case Presentation

**Figure 1:**
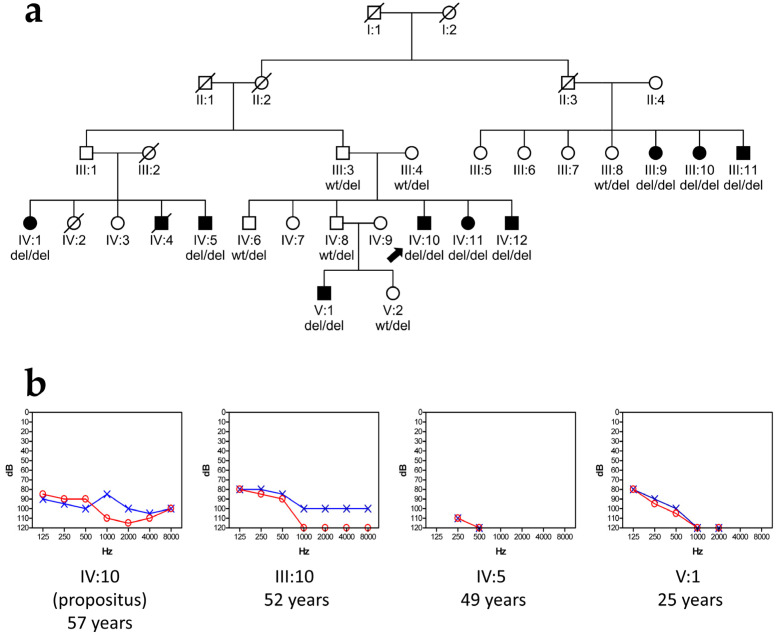
(**a**) The pedigree of the family under study. wt, wild-type allele; del, allele with a 1259 bp deletion. The propositus is indicated by an arrow. (**b**) Air-conduction audiograms of the propositus and three other affected subjects in the pedigree, showing their ages when the test was performed. Red circles, right ear; blue crosses, left ear.

The propositus was a 60-year-old man with congenital deafness from a family of Mayan descent in the Chicán community. He is the first son of parents with normal hearing. He has two affected siblings (59 and 50 years old, respectively) and three siblings with normal hearing (Figure 1a). No clinical signs suggestive of a syndromic condition were identified in the proband or in other family members. Audiological examination included otoscopy, tympanometry, and pure-tone audiometry (testing for air conduction, range of frequencies 250–8000 Hz). Pure-tone audiometry showed a profound bilateral sensorineural hearing loss in the propositus, which was classified as non-syndromic because of the absence of other clinical signs (Figure 1b). The same phenotype was observed in his two affected siblings and in six other hearing-impaired relatives in the extended pedigree (Figure 1a), i.e., congenital profound hearing loss that remains stable across the years. Representative audiograms are shown in Figure 1b. None of the affected subjects complained of vestibular symptoms. They were provided with hearing aids, but their utility has been scarce given the severity and early onset of their hearing losses. Instead, they use their own Chican Yucatecan Maya Sign Language, which has its own linguistic structure in Mayan and a vigesimal numeric system.

Blood samples were collected from the participants in this study, and genomic DNA was extracted by a salting out method and quantified by spectrophotometry using NanoDrop 2000 equipment (Thermo Fisher Scientific, Waltham, MA, USA).

**Figure 2:**
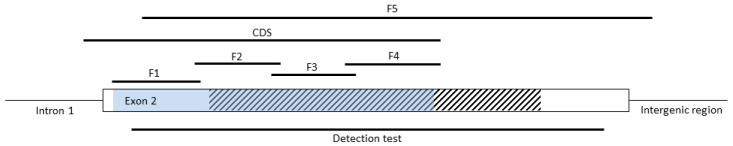
A schematic drawing of *GJB2* exon 2 and adjacent sequences. The coding sequence is shown in blue. The deleted sequences are cross-hatched. Horizontal lines above and below the exon indicate the location of the different amplicons in Table 1.

In order to identify pathogenic variants in the *GJB2* gene (NM_004004.6), we performed PCR on the proband’s DNA by using a pair of primers covering its whole coding DNA sequence (CDS) (amplicon CDS, Figure 2, Table 1 and Table 2). Surprisingly, we did not obtain any amplification product, which suggested the existence of a genetic variant overlapping the sequence of at least one of the primers. To detect this hypothetical variant, four pairs of primers were designed to divide the coding region into four overlapping PCR fragments (amplicons F1-F4, Figure 2, Table 1 and Table 2). Only F1 could be amplified from the proband. We hypothesized that a deletion had occurred, with a breakpoint downstream of F1 (nucleotide 201 of *GJB2* coding region). To determine whether the deletion was intragenic or it may extend far downstream the gene, we amplified fragment F5, spanning from nucleotide 120 within the *GJB2* coding region to position 2112 + 103 in the intergenic region, so that the whole 3′ UTR was contained in the fragment (Figure 2). The expected F5 size in a wild type subject was 2015 bp, but we obtained a PCR product of about 750 bp from the proband, which confirmed he was carrying a large intragenic deletion.

**Figure 3:**
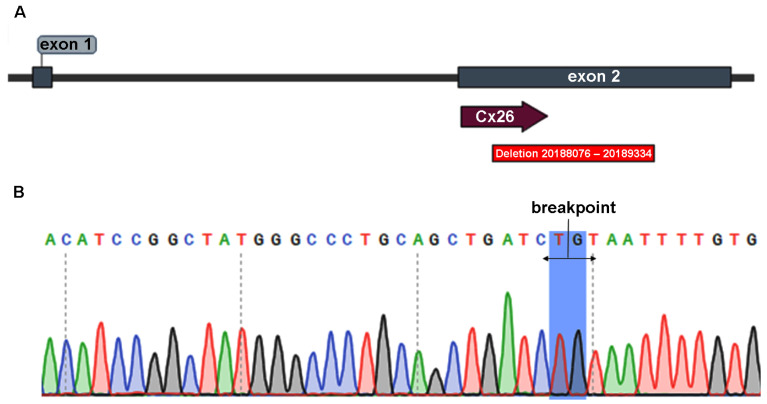
(**A**) The location of the 1259 bp deletion in the *GJB2* gene. Diagram was constructed with SnapGene 6.2.2 software. (**B**) PCR chromatogram showing the breakpoint junction of the 1259 bp deletion.

The PCR-amplified F5 products were treated with ExoSAP-IT PCR Product Cleanup Reagent (Thermo Fisher Scientific, Waltham, MA, USA) and bidirectionally sequenced using the BigDye Terminator v3.1 Cycle Sequencing Kit on the four-capillary Seqstudio analyzer (Applied Biosystems, Waltham, MA, USA) at the DIMYGEN Laboratory S.C.P. Chromatograms were analyzed with BioEdit V.7.2.5 and SnapGene 6.2.2 software, then compared to the reference sequence (NM_004004.6) available in the National Center for Biotechnology Information (NCBI) database (https://www.ncbi.nlm.nih.gov/, accessed on 14 July 2023). The open reading frame (ORF) and its amino acid sequences were then identified using the Open Reading Frame Finder (https://www.ncbi.nlm.nih.gov/orffinder/, accessed on 14 July 2023) from NCBI. The obtained sequence was compared to the reference sequence (NP_003995.2) from NCBI. The precise genomic position of the identified variant was determined using the standard nomenclature recommended by the Human Genome Variation Society (HGVS) version 21.0.2 (https://hgvs-nomenclature.org/stable/, accessed on 14 July 2023). We identified a novel 1259 bp deletion in exon 2 of the *GJB2* gene, starting at nucleotide 248 of the coding region and ending at nucleotide 825 of the 3′-UTR (genomic coordinates in GRCh38, NC_000013.11: g.20188077_20189335del; cDNA coordinates, c.248_*825del), resulting in a loss of 59.0% of the exon (Figure 2 and Figure 3).

**Figure 4:**
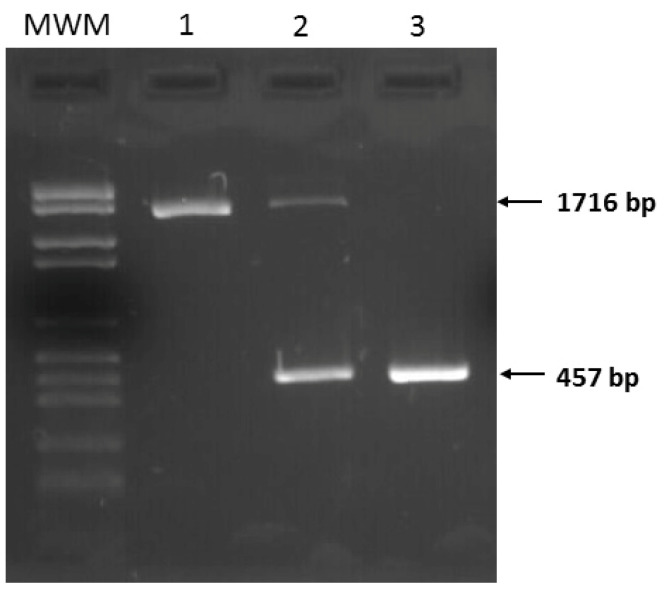
The electrophoresis of the PCR products on a 2% agarose gel stained with GreenSafe Premium (NZY tech, LDA, Lisbon, Portugal), showing the amplified bands corresponding to the three genotypes for the large deletion. The 1716 bp band corresponds to the wild-type allele, and the 457 bp band corresponds to the allele with the deletion. MWM: Molecular weight marker (Roche, Basel, Switzerland). Lane 1: Wild-type genotype (+/+). Lane 2: Heterozygous genotype (+/−); Lane 3: Homozygous deletion (−/−).

Once the precise break points were identified, another PCR assay was designed to easily test for the deletion by amplifying a shorter amplicon (Figure 2, Table 1 and Table 2). This PCR assay generates a product of 1716 bp from the wild type allele and a product of 457 bp from the allele with the deletion (Figure 4). By using this test, we studied the segregation of the deletion in the family, which confirmed that the proband and his two affected siblings were homozygous for the identified deletion, while the parents and two unaffected siblings were heterozygous carriers. Six other hearing-impaired homozygous relatives and two other heterozygous relatives were confirmed in the pedigree. Normal hearing was confirmed in all heterozygous subjects upon audiological evaluation. All family members received genetic counseling based on genetic testing.

The deletion was not found in 153 ethnically matched control subjects with normal hearing (from the same Mexican Mayan community as the propositus). Previous to this work this variant was not reported in the literature or in population databases such as ClinVar (https://www.ncbi.nlm.nih.gov/clinvar, accessed on 15 June 2025), gnomAD (https://gnomad.broadinstitute.org/, accessed on 15 June 2025), ClinGen (https://erepo.clinicalgenome.org/evrepo/, accessed on 15 June 2025), LOVD (https://www.lovd.nl/, accessed on 15 June 2025), and Franklin by Genoox (https://franklin.genoox.com/clinical-db/home, accessed on 15 June 2025). According to the guidelines of The American College of Medical Genetics and Genomics/Association for Molecular Pathology (ACMG/AMP) [24,25,26], this deletion was classified as “pathogenic”.

**Figure 5:**
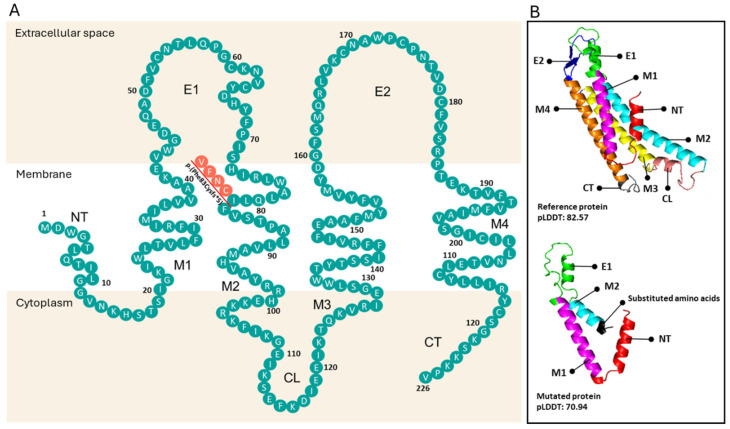
(**A**) Topology of Cx26. The variant p.(Phe83Cysfs*5) is indicated with red lines. (**B**) Cx26 protein monomers as predicted by AlphaFold2 (https://colab.research.google.com/github/sokrypton/ColabFold/blob/main/AlphaFold2.ipynb). **Top**: Reference protein. **Bottom**: Truncated protein resulting from the p.(Phe83Cysfs*5) variant. Transmembrane domains: M1, M2, M3, and M4; extracellular helices: E1 and E2; intracellular loop: CL; N-terminal end: NT; C-terminal end: CT.

The analysis of the coding region showed a frameshift starting at codon 83. As a result, phenylalanine-83 is replaced by cysteine, and a premature stop codon is generated, which would result in the truncation of the protein after a tail of four incorrect residues were added (p.(Phe83Cysfs*5)). To determine how the novel variant affects the conformation of Cx26, 3D structural models of the wild-type and mutant proteins were constructed in AlphaFold2 and visualized with PyMol 2.5.4. A truncated protein composed of only 86 amino acids was predicted (Figure 5A). Although degradation is not predicted to occur, the truncation of the last two-thirds of the protein removes the transmembrane domain 2 (M2), the intracellular loop (CL), the transmembrane domain 3 (M3), the extracellular helix (E2), the transmembrane domain 4 (M4), and the C-terminal end (CT), which are critical for the function of the Cx26 protein (Figure 5B).

## 3. Discussion

In this study, we have identified a novel DFNB1 pathogenic variant, which is unique as large intragenic deletions within exon 2 of *GJB2* have not been reported to date. Large deletions at the DFNB1 locus are known to cause their effects through two different mechanisms of pathogenesis (Table 3): (i) Six deletions do not affect *GJB2* directly, but have been shown to reduce its expression by removing an unidentified cis-regulatory element, presumably located within the common genomic interval deleted in these variants [27,28,29,30,31,32]; (ii) Other deletions directly affect *GJB2* by removing either the whole gene sequence (two deletions) [33,34] or its 5′ part, including about half of the coding sequence (one deletion) [35]. The novel deletion we report here belongs to this second group, although it differs from the others in that it is intragenic and it removes the 3′ part of the coding sequence, presumably leading to produce a truncated polypeptide.

This 1259 bp deletion spans from the first third of the ORF to two-thirds of the 3′-UTR of the last exon of the *GJB2* gene. Since the whole coding sequence of the *GJB2* gene is contained within the last exon, it is expected that mRNAs resulting from variants causing a frameshift and leading to a premature stop codon escape nonsense-mediated decay. This would lead to the synthesis of truncated proteins [36], which has been confirmed in several studies [37,38].

Multiple pathogenic and likely pathogenic variants in the *GJB2* gene have been identified that produce truncated proteins similar to the p.(Phe83Cysfs*5) variant. These proteins lack one or more of the transmembrane segments and extracellular loops, impairing the folding and oligomerization of Cx26 [38]. Particularly, the E1 and E2 domains are highly conserved and each contains three pairs of cysteines, which are crucial for channel formation. These cysteines provide multiple anchoring points between E1 and E2 through disulfide bond formation. These bonds are critical for the proper folding of Cx26 and the formation of a functional coupling structure, prerequisites for channel formation. Additionally, connexon pairing occurs through hydrogen bonds (non-covalent interactions), with four bonds for E1-E1 interactions and six for E2-E2 interactions [39].

Truncated proteins have also been observed to have poor membrane trafficking/orientation and tend to be retained in intracellular compartments such as the endoplasmic reticulum, leading to a loss of function. Functional studies have shown that some of these truncated proteins fail to reach the cell surface and accumulate in the endoplasmic reticulum, such as c.235del (p.(Leu79Cysfs3)), c.465T>A (p.(Tyr155*)), c.572delT (p.(Phe191Serfs5)), and c.631–632delGT (p.(Cys211Leufs*5)) [37,38].

The high genetic heterogeneity of hearing loss complicates the molecular genetic diagnosis of these disorders. The approach taken to address this complexity is the identification of high-frequency pathogenic variants in a limited set of genes. Currently, screening for the *GJB2* gene is considered a standard and first-line approach in diagnosing genetic hearing loss. Our finding is of concern to *GJB2* screening methods that rely primarily on sequencing one or several amplicons covering only its coding region, as intragenic deletions like the one we report here could be overlooked in heterozygous subjects. If they carried a pathogenic point variant in the other allele (e.g., c.35delG), this deletion could make it look as homozygous. This underscores the need of studying the segregation of variants in the parents of affected subjects. Massively parallel sequencing including analysis of copy number variations should fix this problem, but laboratories that have no easy access to these methodologies should consider the need to perform Sanger sequencing on longer amplicons, encompassing both the coding sequence and the whole 3′-UTR.

Pathogenic variants in this gene remain the most common cause of non-syndromic hearing impairment across populations. Various studies have observed that different pathogenic variants in the *GJB2* gene have high frequency in specific populations [40]. In the Maya Mexican population, there was no prior data on pathogenic variants associated with hearing impairment. The identification of this variant and its implication in congenital hearing loss underscores the importance of conducting detailed genetic studies in specific populations to better understand genetic diversity and its clinical effects.

## Figures and Tables

**Table 1:** The primers that were used in this study.

Amplicons	Sequences	Position in *GJB2* CDS *	
		Start	End
CDS	CDSA: 5′ ACCTGTTTTGGTGAGGTTGTGT 3′	−22 − 140	−22 − 119
	CDSB: 5′ TGAGCACGGGTTGCCTCATC 3′	745	726
F1	F1A: 5′ CAAACCGCCCAGAGTAGAAG 3′	−20	−1
	F1B: 5′ GTGATCGTAGCACACGTTCTTG 3′	201	180
F2	F2A: 5′ CCAGGCTGCAAGAACGTGTG 3′	172	191
	F2B: 5′ TCGAAGATGACCCGGAAGAA 3′	440	421
F3	F3A: 5′ TCGAGGAGATCAAAACCCAGAAG 3′	353	375
	F3B: 5′ GCAAATTCCAGACACTGCAATCA 3′	606	584
F4	F4A: 5′ GCCTTGTCCCAACACTGTGGACT 3′	516	538
	CDSB: 5′ TGAGCACGGGTTGCCTCATC 3′	745	726
F5	F5A: 5′ AAAGGAGGTGTGGGGAGATGAG 3′	120	141
	F5B: 5′ GGCAACTTACCCATTGGTGTTAT 3′	2112 + 103	−2112 + 81
Detection test	DTA: 5′ CGCATTATGATCCTCGTTGTG 3′	94	114
	DTB: 5′ AGGCTGAAGGGGTAAGCAAAC 3′	1809	1789

* Nucleotide position refers to the coding sequence within exon 2 of *GJB2*, the “A” of the translation initiation codon being considered as +1.

**Table 2:** PCR programmers for the amplicons that were used in this study.

Amplicons	PCR Programs
CDS	96 °C, 5 min/94 °C for 40 s, 60 °C for 40 s, 72 °C for 1 min (30 cycles)/72 °C, 7 min
F1–F4	96 °C, 5 min/94 °C for 40 s, 60 °C for 40 s, 72 °C for 30 s (30 cycles)/72 °C, 7 min
F5	94 °C for 40 s, 68 °C (−1 °C/cycle) for 40 s, 72 °C for 90 s (5 cycles)/94 °C for 40 s, 63 °C for 40 s, 72 °C for 90 s (30 cycles)/72 °C, 10 min
Detection test	96 °C, 5 min; 94 °C for 40 s, 59 °C for 40 s, 72 °C for 1 min 45 s (30 cycles); 72 °C, 7 min

**Table 3:** Large deletions at the DFNB1 locus.

Deletion Name	Size	Chr13 *		Affected Genes	Reference
		Start	End		
del(920 kb)	920 kb	19,558,829–19,569,782 (breakpoint unknown)	20,489,408–20,491,267 (breakpoint unknown)	*MPHOSPH8, PSPC1, ZMYM5, ZMYM2, GJA3, GJB2, GJB6, CRYL1*	[31]
del(*GJB6*-D13S1830)	309 kb	20,223,038	20,531,806	*GJB6, CRYL1*	[25]
del(*GJB6*-D13S1854)	232 kb	20,228,588	20,460,629	*GJB6*, *CRYL1*	[26]
del(200 kb)insATTATA	200 kb	20,361,160	20,561,391	*CRYL1*	[27]
del(179 kb)	179 kb	20,347,572	20,526,976	*CRYL1*	[28]
del(131 kb)	131 kb	20,365,205	20,496,559	*CRYL1*	[29]
del(125 kb)	125 kb	20,398,370	20,523,823	*CRYL1*	[30]
del(*GJB2*-D13S175)	101 kb	20,182,882	20,284,255	*GJB2, GJB6*	[32]
del(8 kb)	8 kb	20,189,271	20,197,662	*GJB2*	[33]
del(1259 bp)	1259 bp	20,188,077	20,189,335	*GJB2* (intragenic)	This work

* Coordinates on human reference genome GRCh38/hg38.

## Data Availability

Data on the pathogenic variant that is reported in this study are available in ClinVar (accession number VCV003069173.2).

## Abbreviations

The following abbreviations are used in this manuscript:

3′-UTR	3′ Untranslated Region
CDS	Coding DNA Sequence
ORF	Open Reading Frame
